# Improved Methods to Produce Tissue-Engineered Skin Substitutes Suitable for the Permanent Closure of Full-Thickness Skin Injuries

**DOI:** 10.1089/biores.2016.0036

**Published:** 2016-11-01

**Authors:** Danielle Larouche, Laurence Cantin-Warren, Maxime Desgagné, Rina Guignard, Israël Martel, Akram Ayoub, Amélie Lavoie, Robert Gauvin, François A. Auger, Véronique J. Moulin, Lucie Germain

**Affiliations:** ^1^ Département de Chirurgie, Faculté de Médecine, Centre de Recherche en Organogénèse Expérimentale de l'Université Laval/LOEX, Université Laval, Québec, Canada.; ^2^ Centre de Recherche du CHU de Québec—Université Laval, Axe Médecine Régénératrice, Québec, Canada.; ^3^ Centre Québécois sur les Matériaux Fonctionnels (CQMF), Québec, Canada.

**Keywords:** autologous, regenerative medicine, skin equivalent, tissue culture, tissue therapy, transplants

## Abstract

There is a clinical need for skin substitutes to replace full-thickness skin loss. Our group has developed a bilayered skin substitute produced from the patient's own fibroblasts and keratinocytes referred to as Self-Assembled Skin Substitute (SASS). After cell isolation and expansion, the current time required to produce SASS is 45 days. We aimed to optimize the manufacturing process to standardize the production of SASS and to reduce production time. The new approach consisted in seeding keratinocytes on a fibroblast-derived tissue sheet before its detachment from the culture plate. Four days following keratinocyte seeding, the resulting tissue was stacked on two fibroblast-derived tissue sheets and cultured at the air–liquid interface for 10 days. The resulting total production time was 31 days. An alternative method adapted to more contractile fibroblasts was also developed. It consisted in adding a peripheral frame before seeding fibroblasts in the culture plate. SASSs produced by both new methods shared similar histology, contractile behavior *in vitro* and *in vivo* evolution after grafting onto mice when compared with SASSs produced by the 45-day standard method. In conclusion, the new approach for the production of high-quality human skin substitutes should allow an earlier autologous grafting for the treatment of severely burned patients.

## Introduction

The standard surgical treatment for the permanent closure of large full-thickness skin wounds that can occur following acute trauma or surgical intervention consists in replacing tissue loss with skin autografts harvested from an uninjured donor site on the patient. However, when the skin loss exceeds 50% of the total body surface area of the patient, donor site availability for harvesting autografts becomes limited. Progress in tissue engineering has led to the development of technologies, allowing the production of skin substitutes. These substitutes can be either epidermal or dermal substitutes or bilayered skin substitutes.

Tissue-engineered bilayered skin substitutes are composed of both a dermis and an epidermis. The ideal bilayered skin substitute should be easy to handle, must permanently ensure the skin barrier function, and should not induce a host immune rejection. Some models of bilayered skin substitutes produced in the laboratory have been reported to allow permanent coverage of full-thickness wound, to reduce the need for harvesting autografts, and are indicated as an adjunct treatment for massive burns.^1–3^

The self-assembly approach developed at the LOEX^4,5^ allows for the reconstruction of a fully autologous bilayered skin substitute. This method is based on the capability of cells to form an organized three-dimensional tissue without using any exogenous scaffold or biomaterial. The resulting skin tissue shares many properties with native human skin and minimizes the host response after transplantation. The self-assembly method generates a highly functional and mechanically stable skin substitute^6^ preserving epithelial stem cells,^7^ which is suitable for autologous grafting in humans.^8^

The first published self-assembly method involves a 45-day production period that includes the fabrication of the dermal component (28 days), the seeding of keratinocytes followed by a 7-day submerged culture period, and maturation at the air–liquid interface for an additional 10 days.^9,10^

The aim of the present study was to reduce the production time of Self-Assembled Skin Substitute (SASS) and to standardize the manufacturing protocol for clinical production purposes. The new method only requires a 31-day production period. The resulting SASSs are equivalent in terms of histological properties, contractility, and *in vivo* evolution compared with SASSs produced with the 45-day reference method.

## Materials and Methods

### Cell populations

The study was approved by the institutional animal care and use committee and by the institutional committee for the protection of human subjects. The procedures followed were in accordance with the Helsinki Declaration of 1975.

### Cell isolation and culture

Human keratinocytes and dermal fibroblasts were isolated from 1 newborn and 11 adult (18 to 46 years old) human skin samples as previously described.^10^ Dermal fibroblasts were cultured in fibroblast medium (Dulbecco–Vogt modified Eagle medium [Corning] supplemented with 10% fetal bovine serum [FBS; Seradigm], 100 U/mL penicillin [Pharmaceutical Partners of Canada, Inc.], and 25 μg/mL gentamycin [Galenova]). Keratinocytes were grown on a feeder layer of irradiated human fibroblasts^11^ and cultured in keratinocyte medium (Dulbecco–Vogt modified Eagle medium: Ham's F12, ratio 3:1, 24.3 μg/mL adenine [Corning] supplemented with 5 μg/mL insulin [Sigma-Aldrich], 1.1 mM hydrocortisone [Novapharm], 0.212 μg/mL isoproterenol hydrochloride [Sandoz], 5% bovine Fetal Clone II serum [HyClone], 10 ng/mL human epidermal growth factor [R&D Systems], 100 U/mL penicillin, and 25 μg/mL gentamycin). For tissue production, cells were used at passage three for keratinocytes and passage two to six for fibroblasts.

### Skin substitute production

The tissue-engineered skin methods presented here derive from the SASS method described earlier,^10^ here after referred to as SASS-1. In this study, tissue-engineered skin substitutes produced by three adaptations of the SASS-1 method were compared: the method referred to as SASS-2 (reference method, previously presented in Ref.^6^), SASS-3, and SASS-4. Each proposed method was performed 6–10 times in triplicate using different combinations of fibroblasts and keratinocytes, of which four combinations were donor matched.

Fibroblasts were seeded at a density of 4 × 10^3^ cells/cm^2^ in 85 cm^2^ Nunc™ Omnitray™ tissue culture plate with a removable lid (Fisher Scientific) and cultured in fibroblast medium containing 50 μg/mL ascorbic acid (Galenova) to promote extracellular matrix production.

**FIG. 1.**
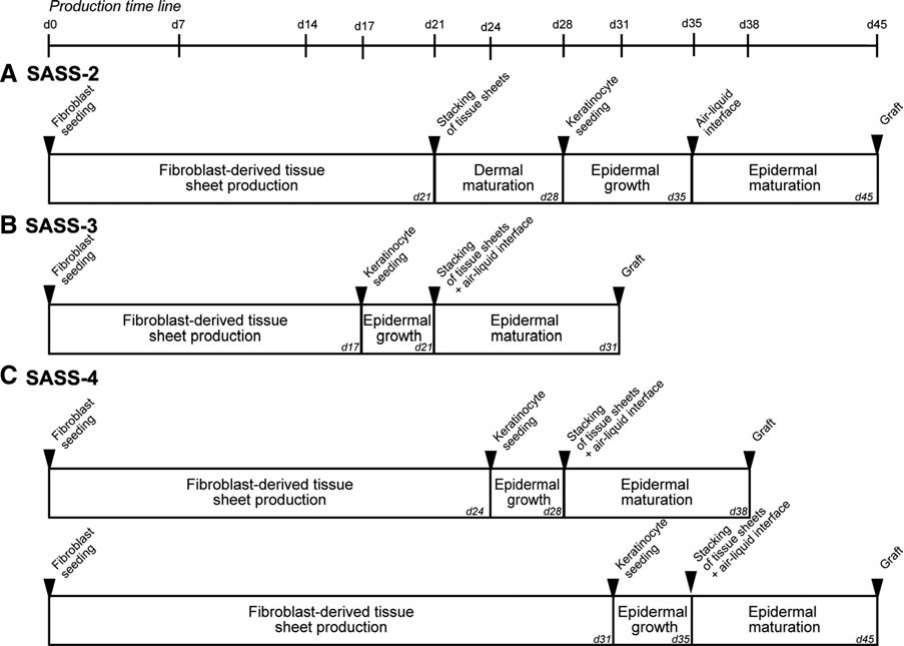
Schematic representation of the timeline of the main steps in the production of SASS-2 **(A)**, SASS-3 **(B)**, and SASS-4 **(C)**. SASS, Self-Assembled Skin Substitute.

The method SASS-2 consists of placing a custom-made frame (inner dimensions: 46 × 76 mm, outer dimensions: 62 × 99 mm) cut out of Ahlstrom grade 237 filter paper (Fisher Scientific) onto a 21-day fibroblast-derived tissue sheet still attached to the bottom of the culture plate. Tissue surrounding the frame is folded thereon. The frame is grasped with forceps, and the tissue is carefully detached and stacked on a subsequent tissue sheet. Surrounding tissue is again folded onto the frame, and the second tissue sheet is detached. The procedure is repeated with a third fibroblast-derived tissue sheet. Then, the stack of three sheets is attached to the paper frame using LIGACLIPS^®^ (Ethicon Endo-Surgery) and placed in a 150 mm diameter culture Petri dish (Corning). For the first 24 h, a surgical sponge (Merocel^®^; Medtronic, Instruments Ophtalmiques INNOVA) is installed on the top of stacked sheets and held in place by stainless steel weights. After 7 days of culture in fibroblast medium containing 50 μg/mL ascorbic acid, 0.9 to 2 × 10^5^ keratinocytes/cm^2^ are seeded within a stainless steel seeding mold (inner dimensions: 48 × 76 mm, outer dimensions: 50 × 78 mm) placed on the top of the stacked tissue sheets. After 7 days of culture submerged in keratinocyte medium containing 50 μg/mL ascorbic acid, the resulting tissue is detached from the bottom of the culture plate and raised on a custom-made frame (inner dimensions: 46 × 76 mm, outer dimensions: 96 × 113 mm) cut in filter paper (Ahlstrom 237) or onto a 100 × 100 mm polypropylene membrane (Spectra/Mesh^®^Woven polypropylene membrane filters, mesh opening: 500 μm, open area: 2%, thickness: 610 μm, Spectrumlabs.com). The construct is then transferred on a custom-made acrylic support to maintain the tissue at the air–liquid interface in a 150 mm diameter culture Petri dish. The tissue is further cultured for 10 days at the air–liquid interface in keratinocyte medium exempt of epidermal growth factor and containing 50 μg/mL ascorbic acid. The resulting skin substitute is referred to as SASS-2. The total production time is 45 days (Fig. 1A).

The method SASS-3 consists in seeding 0.9 to 2 × 10^5^ keratinocytes/cm^2^ directly onto the top of a 17-day fibroblast-derived tissue sheet, produced as described above, still attached to the bottom of the culture plate. The tissue is then cultured in keratinocyte medium containing 50 μg/mL ascorbic acid, and medium was changed once a day. After 4 days, a custom-made filter paper frame (peripheral anchorage inner dimensions: 46 × 76 mm, outer dimensions: 62 × 99 mm, Ahlstrom grade 237 filter paper) is placed onto the tissue sheet containing keratinocytes and fibroblasts. The tissue adheres to the frame. The thin strip (2–4 mm) of tissue surrounding the frame is folded thereon without covering the inside tissue. Both extremities of the frame are grasped with forceps, and the tissue is progressively raised. Once detached, the tissue is stacked onto a 21-day fibroblast-derived tissue sheet. Surrounding tissue is folded onto the frame without covering the inside tissue, and the second tissue sheet is detached. The procedure is repeated with another 21-day fibroblast-derived tissue sheet, and the three-sheet stacked tissue is fixed to the frame using LIGACLIPS (Ethicon Endo-Surgery). The construct is transferred onto a polypropylene membrane laid on a custom-made acrylic support to hold the tissue at the air–liquid interface in a 150 mm diameter culture Petri dish and further cultured 10 days at the air–liquid interface as described above. The resulting skin substitute is referred to as SASS-3. The total production time is 31 days (Fig. 1B).

According to the patient's cells, dermal fibroblasts can sometimes induce contraction of unanchored tissue sheets when keratinocytes were added on the sheet. To circumvent this problem, an alternative approach referred to as method SASS-4 was developed. It consists of placing a custom-made frame (inner dimensions: 57 × 84 mm, outer dimensions: 73 × 102 mm) cut out of filter paper (Ahlstrom grade 610 filter paper [Fisher Scientific]) into the culture plate before seeding the fibroblasts.^12,13^ Thus, cells grow in the culture plate and adhere to the peripheral paper anchorage. Cells are cultured in fibroblast medium containing 50 μg/mL ascorbic acid for typically 24–31 days. Then, 0.9 to 2 × 10^5^ keratinocytes/cm^2^ are seeded directly onto the top of an anchored fibroblast-derived tissue sheet still attached to the bottom of the culture plate. The tissue is then cultured in keratinocyte medium containing 50 μg/mL ascorbic acid, and the medium was changed once a day. After 4 days, the tissue is carefully detached and stacked with two other anchored fibroblast tissue sheets. The remaining steps are identical to those of the method SASS-3. The resulting skin substitute is referred to as SASS-4. The total production time is 38–45 days (Fig. 1C).

All cultures and skin substitutes were kept at 37°C in a humidified incubator containing 8% CO_2_, and the culture medium was changed three times per week except when otherwise indicated.

### Contraction kinetic on agar-gelled substrate

The structural stability of the skin substitutes was assessed at the end of the culture period (after 10 days of culture at the air–liquid interface) by measuring tissue contraction with the method of agar-gelled substrate as described.^6^ Surface area of the skin substitute was measured over time (0, 1, 2, 4, 6, 8, 24, and 48 h) on scale-calibrated pictures using ImageJ^®^ software (NIH). The initial area of the die cut (9 cm^2^) was determined as 100%, and all subsequent measurements were expressed as a ratio to this value. The experiment was repeated twice using newborn or adult cells each time in triplicate.

### Grafting on athymic mice

SASSs were grafted on athymic male nu/nu mice (42 days old) (Charles River Laboratories) as described.^14^ After detaching the skin substitute from its paper frame anchorage, sterile nonadherent gauze was placed on top to facilitate the transfer to the graft bed. Six mice per condition were grafted. Fusenig's silicone chambers were removed after 21 days. For each condition, mice were sacrificed 21 (3 mice) and 90 (3 mice) days after grafting. Skin substitute samples (before grafting, 21 and 90 days after grafting) were processed for histological and immunofluorescence analysis. The experiment was repeated three times, each time with SASS-2, SASS-3, and SASS-4 produced with two donor-matched keratinocytes and fibroblasts.

### Histological and immunofluorescence analysis

Tissue biopsies were fixed overnight in HistoChoice^®^ (Amresco) and embedded in paraffin. Five micrometer-thick sections were stained with Masson's trichrome using Weigert's hematoxylin, fuchsin-ponceau, and aniline blue.

For immunodetection, biopsies were embedded in Tissue-Tek OCT Compound (Sakura Finetek) and frozen in liquid nitrogen. Immunofluorescence assays were performed on 5 μm-thick cryosections fixed with acetone (10 min at −20°C) as previously described.^14^ Cell nuclei were counterstained with Hoechst reagent 33258 (Sigma-Aldrich). The antibodies used were as follows: mouse monoclonal anti-K10 clone RKSE60 (Cedarlane), anti-human filaggrin (Abcam), anti-human K19 clone A53-B/A227 (gift from U. Karsten, Institute of Biological Sciences, University of Rostock, Germany), anti-integrin alpha-3 (VM2) clone HB-8530 (ATCC), anti-laminin-5 (alpha-3 subunit) conjugated to fluorescein isothiocyanate (FITC) (gift from P. Rouselle, IBCP, Lyon), anti-human Ki67 (Pharmingen), anti human leukocyte antigen A, B, C (HLA-ABC), conjugated to FITC (EMD Millipore), and rabbit anti-human type IV collagen (gift from J.A. Grimaud, Pasteur Institute, Lyon, France).

### Transmission electron microscopy

Samples were processed and pictures were acquired as described.^15^ To evaluate the maturation of the basement membrane, a score representing the estimated percentage of the dermoepidermal junction with *lamina densa* (1 = 0–10%, 2 = 11–20%, 3 = 21–30%, 4 = 31–40%, 5 = 41–50%, 6 = 51–60%, 7 = 61–70%, 8 = 71–80%, 9 = 81–90%, 10 = 91–100%) was given for SASS-2, SASS-3, and SASS-4 specimens from at least four independent experiments.

## Results

The tissue engineering method previously described to produce SASS-2^6^ was compared with two new methods (SASS-3, SASS-4) allowing for a shorter production time. All three types of substitutes have been successfully produced with most keratinocyte and fibroblast populations tested, except with cells from a 30-year-old female donor where only SASS-2 and SASS-4 have been successfully produced. In this case, the addition of the donor-matched keratinocytes onto the unanchored fibroblast-derived sheet (SASS-3) induced contraction resulting in unusable shrunk tissue.

### Macroscopic appearance and histological analysis of SASS cultured *in vitro*

**FIG. 2.**
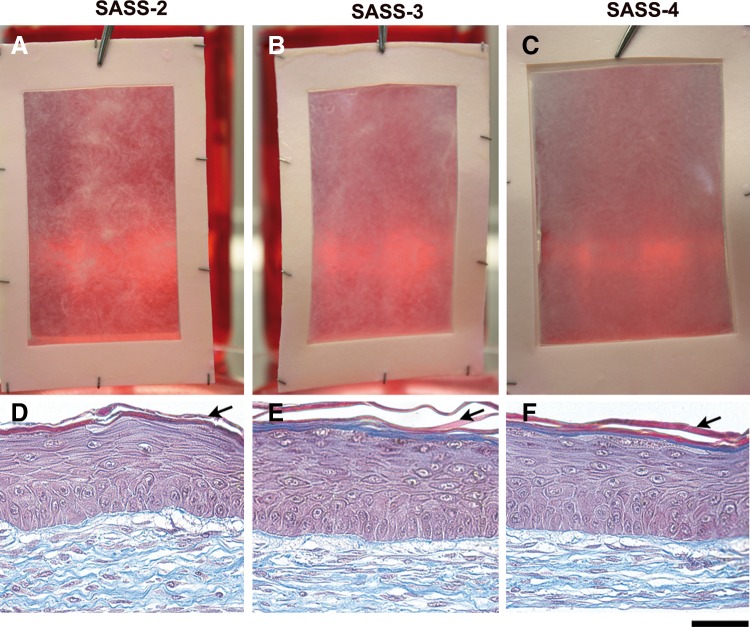
Macroscopic and histological analysis of SASS produced *in vitro*. Representative macroscopic **(A–C)** and histological **(D–F)** results of SASS-2 (left panels), SASS-3 (center panels), and SASS-4 (right panels) produced with keratinocytes and fibroblasts from the same donor. Arrows point out squame accumulation. Scale bar: **(A–C)**, 167 mm; **(D–F)**, 50 μm.

**FIG. 3.**
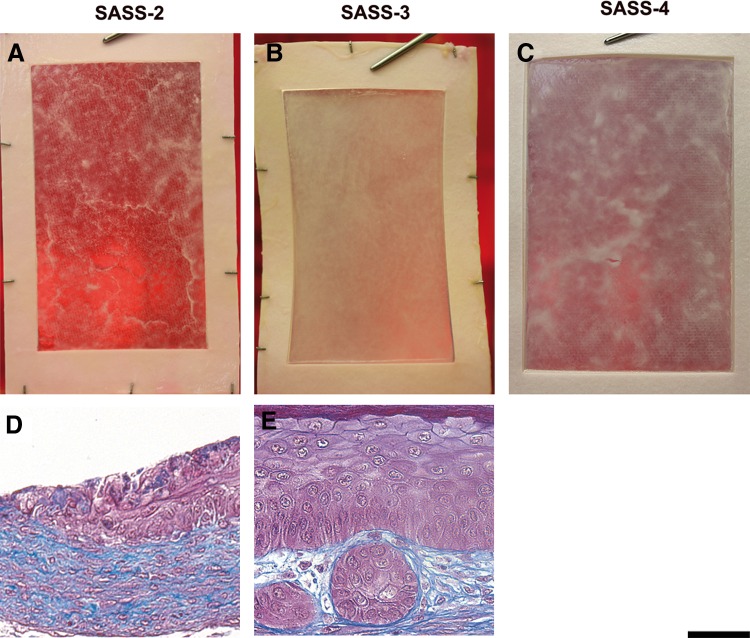
Macroscopic and histological analysis of SASS produced *in vitro*. Representative macroscopic **(A–C)** and histological **(D, E)** results of SASS-2 (left panels), SASS-3 (center panels), and SASS-4 (right panel) produced with keratinocytes and fibroblasts from the same donor (different from the donor of Fig. 1). Representative histological aspect of an area appearing brighter macroscopically **(D)** and an epithelial cell inclusion **(E)**. Scale bar: (**A–C)**, 167 mm; (**D, E)**, 50 μm.

After 10 days of *in vitro* maturation at the air–liquid interface, SASS-2, SASS-3 and SASS-4 appeared macroscopically as a tissue that resembled human skin (Fig. 2A–C, respectively). Of note, the surface of SASS-3 and SASS-4 was more homogeneous compared to that of SASS-2 (Fig. 2, compare B and C with A). Surface uniformity varied between cell donors. Areas appearing denser or brighter were observed (Fig. 3, compare A with B) in a few cases. Again, these irregularities were more common in SASS-2 compared to SASS-3 and SASS-4 for a given combination of keratinocytes and fibroblasts (Fig. 3, compare B and C with A).

Histological analyses of SASS-2, SASS-3, and SASS-4 revealed the presence of epidermis covering the dermal component at 10 days of culture at the air–liquid interface for all tested conditions. The four typical layers of human epidermis (*stratum basale*, *spinosum, granulosum,* and *corneum*) were observed in denser areas of SASS, indicating a fully differentiated epidermis (Fig. 2D–F, respectively). For less homogeneous SASS, areas that appeared brighter macroscopically were typically associated with a lower number of keratinocyte layers and the absence of a *stratum corneum* (Fig. 3D), while denser areas were rather associated to a fully differentiated epidermis with squame accumulation (as observed in Fig. 2D–F, arrows).

Some epithelial cell inclusions were seldom observed within the dermal component of SASS (Fig. 3E). Some of these structures were in contact with the epidermis forming invaginations (data not shown). The occurrence of epithelial inclusion was higher within SASS-3 and SASS-4 compared with SASS-2.

### Immunofluorescence analysis

**FIG. 4.**
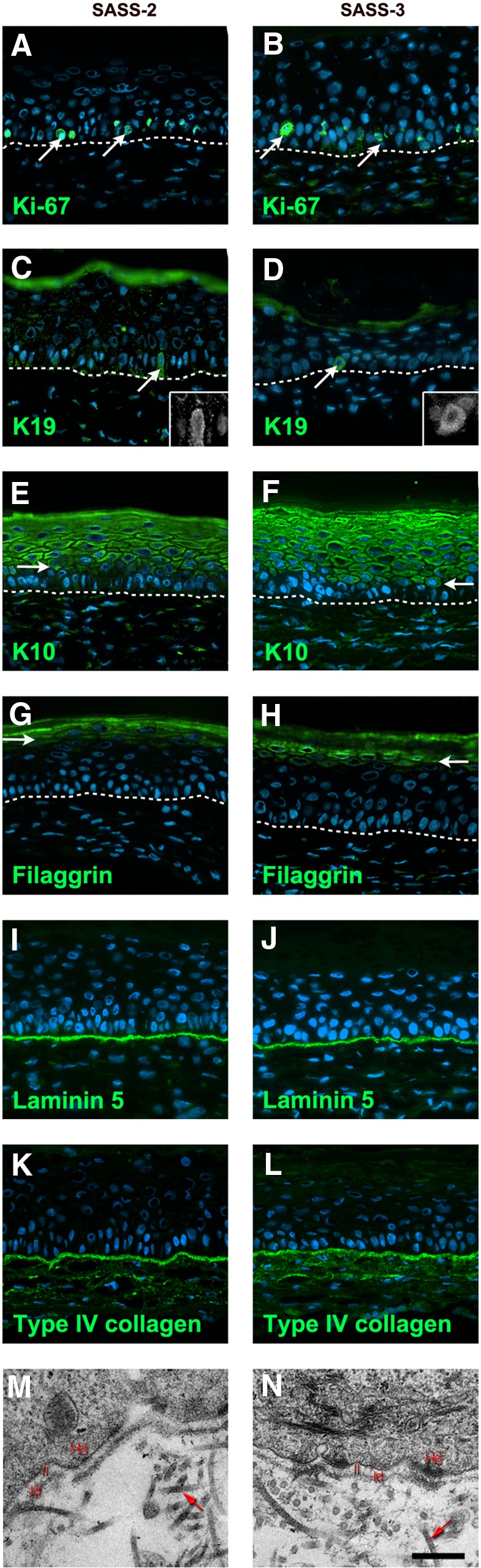
Analysis of skin markers and of the ultrastructure of SASS matured *in vitro*. Representative pictures of SASS-2 (left panels) and SASS-3 (right panels) produced with keratinocytes and fibroblasts immunolabeled for the detection of Ki-67 (**A, B,** arrows), K19 (**C, D,** arrows, inset: high magnification of the cell pointed by the arrow), K10 (**E, F,** arrows point toward the first labeled cell layer), filaggrin (**G, H,** arrows point toward the first labeled cell layer), laminin-5 **(I, J)**, and type IV collagen **(K, L)**. Representative transmission electron microscopy observations **(M, N)**. Cell nuclei were stained in blue with Hoechst reagent **(A–L)**. Dotted line indicates the dermoepidermal junction. Red arrows point to collagen fibers **(M, N)**. Hd, hemidesmosomes; ll, *lamina lucida*; ld, *lamina densa*. Scale bar: **(A–L)**, 50 μm; **(M, N)**, 310 nm.

The proliferation marker Ki-67 was detected in the nucleus of many cells within the *stratum basale* of SASS-2, SASS-3, and SASS-4 (Fig. 4A, B, arrows, and data not shown), indicating that keratinocytes proliferate in the epidermis.

The expression of skin differentiation markers (K19, K14, K10, filaggrin) was similar between SASS-2, SASS-3 (Fig. 4), and SASS-4 (data not shown). Keratin (K) 19 immunostaining was performed to evaluate the persistence of stem cells and confirmed the presence of a small subset of basal keratinocytes expressing K19 (Fig. 4C, D, arrows). Basal cells expressed K14 and integrin alpha-3 (data not shown), whereas K10 expression was restricted to the suprabasal layers (Fig. 4E, F, arrows). Filaggrin, a protein associated to the cornified envelope, was detected in the upper cell layers of the epidermis (Fig. 4G, H, arrows). Laminin-5 and type IV collagen, two basement membrane components, were detected as a continuous line at the level of the dermoepidermal junction within SASS-2, SASS-3, and SASS-4 (Fig. 4I–L).

Ultrastructural analysis confirmed the presence of typical structures of the basement membrane such as *lamina densa*, *lamina lucida*, and hemidesmosomes for SASS-2, SASS-3, and SASS-4 (Fig. 4M, N and data not shown). The formation of the basement membrane tended to be more advanced in SASS-3 and SASS-4 than in SASS-2 (Table 1).

**Table 1.** **Maturation of the Basement Membrane Within SASS After 10 Days of Culture at the Air–Liquid Interface**

	Mean (SD)	Median	Min	Max
SASS-2 (*n* = 4)	3.8 (2.1)	3.5	2	6
SASS-3 (*n* = 4)	6.0 (3.2)	6.5	2	9
SASS-4 (*n* = 4)	6.0 (2.7)	7.0	2	8

The score assigned represents the estimated percentage of the dermoepidermal junction with *lamina densa* (1 = 0–10%, 2 = 11–20%, 3 = 21–30%, 4 = 31–40%, 5 = 41–50%, 6 = 51–60%, 7 = 61–70%, 8 = 71–80%, 9 = 81–90%, 10 = 91–100%). SASS-3 and SASS-4 were compared to SASS-2 using bilateral Student's *t*-test, and no significant difference was found (*p* ≥ 0.05).

SASS, Self-Assembled Skin Substitute; SD, standard deviation.

### Tissue contraction of SASS

**FIG. 5.**
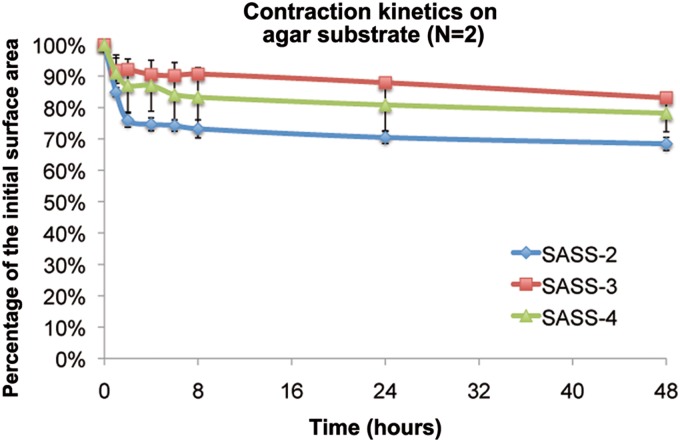
Contractile behavior of SASS matured *in vitro.* SASS-2 (blue curve), SASS-3 (red curve), and SASS-4 (green curve) were placed on an agar substrate, and the surface area of the skin substitutes was measured over time to obtain contraction kinetic curves. N: number of independent experiments performed in triplicate.

The final SASS surface area available for grafting depends on the contraction of the tissue after detachment from the peripheral anchorage at the end of the culture. The structural stability of the skin substitutes after detachment from the anchorage was evaluated *in vitro* over a period of 2 days. Results showed that the contraction kinetic displayed the characteristic exponential decay profile of tissue-engineered skin produce by self-assembly,^6^ independently of the method used for fabrication (Fig. 5). Most of the contraction occurred within 2 h. SASS-2 tended to contract more than SASS-3 and SASS-4 (Fig. 5).

### *In vivo* maturation of SASS after grafting onto athymic mice

**FIG. 6.**
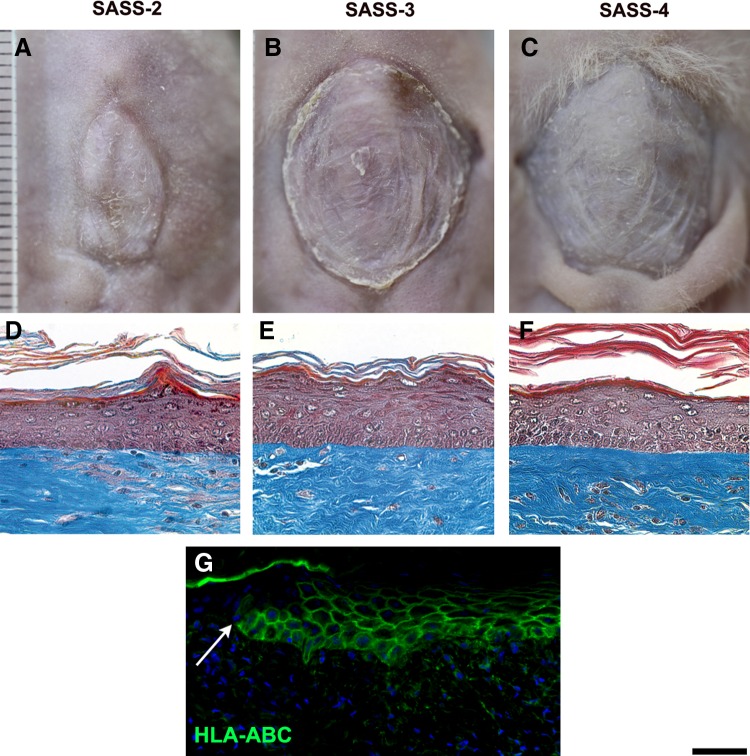
Macroscopic aspect, histological analysis, and immunolabeling for the human histocompatibility complex HLA-ABC of SASS after grafting. Representative macroscopic **(A–C)** and histological **(D–F)** and immunofluorescence **(G)** results of SASS-2 (left panels and **G**), SASS-3 (center panels), and SASS-4 (right panels) produced with keratinocytes and fibroblasts from the same human donor 90 days after grafting onto athymic mice. The staining of the human histocompatibility complex (HLA-ABC) confirmed the human nature of the graft **(G)**. The arrow pointing toward the junction between mouse and human epithelium. Cell nuclei were stained in blue with Hoechst reagent. Scale bar: **(A–C)**, 5 mm; **(D–G)**, 50 μm.

To validate engraftment capacity and permanent integration, SASS-2, SASS-3 and SASS-4 were transplanted on athymic mice for 90 days. Complete take of all grafts to the wound bed was observed. The epithelium was present on all grafts for the entire duration of the experiment, indicating the preservation of functional epithelial stem cells within the skin substitute and long-term epithelial regeneration. During the 21-day period of *in vivo* maturation in the Fusenig's chamber, SASS-2, SASS-3, and SASS-4 surface areas remained stable (data not shown). Three months after grafting, SASS-2, SASS-3 and SASS-4 appeared macroscopically as a uniform and smooth opaque tissue much alike human skin (Fig. 6A–C).

Histological analysis revealed that the evolution of SASS-2, SASS-3, and SASS-4 was similar; the epidermis was well organized with all histological layers present 90 days after grafting (Fig. 6D–F). The persistence of human cells after grafting was confirmed by labeling the human histocompatibility complex HLA-ABC (Fig. 6G).

## Discussion

In this study, alternative methods to produce high-quality autologous human skin substitutes suitable for the permanent coverage of full-thickness skin wounds were proposed. The three designs derive from the self-assembly approach originally presented by our team for blood vessels,^5^ followed by skin in 1999.^9^ The proposed modifications allow adaptation of the manufacturing process that takes into account the patient variability of intrinsic fibroblast properties such as contractile potential and extracellular matrix secretion. Method SASS-3 should be prioritized because it allows the production of skin substitutes in 31 days, which is 2 weeks less than the previous 45 days required for SASS-2, while SASS-4 method manages contractile fibroblasts.

Intrinsic contractile properties of fibroblasts as well as their ability to secrete extracellular matrix vary between donors (personal observation). It happens that unanchored fibroblast-derived tissue sheets spontaneously detached from the culture plate after being in culture. Also, the addition of keratinocytes onto an unanchored fibroblast sheet sometimes induces contraction of the sheet followed by their spontaneous detachment and further tissue shrinkage. The SASS-4 method was designed to circumvent this problem by adding a peripheral anchorage to limit spontaneous tissue contraction. Our results are consistent with the limited tissue contraction observed with anchored adipose tissue sheets or myofibroblast-derived tissue sheets.^12,16^

For SASS production to treat a patient, fibroblasts are used from passage 2. To determine the potential of fibroblasts to generate tissues that resist to manipulation and contraction, a dozen of fibroblast-derived tissue sheets can be prepared from passage 1 fibroblasts to anticipate the behavior of cells, to adjust the optimal culture time, and to orient the choice of the production method to use thereafter.

Epithelial cell inclusions sometimes observed within SASS-2, SASS-3, and SASS-4 were reported to occur in other models of skin substitutes cultured *in vitro.*^17–19^ In all instances, inclusions disappear following skin graft integration and maturation and are not considered to be clinically significant.^17–19^

The quality of the basement membrane and its adequate ultrastructure in SASS may allow for the stem cell preservation since the basement membrane is involved in the maintenance of epithelial stem cells.^20,21^ In our study, we observed that the formation of the basement membrane was more advanced in SASS-3 and SASS-4 compared with SASS-2. The direct seeding of keratinocytes onto a fibroblast-derived tissue sheet still attached to the bottom of the culture plate ensures an optimal distribution of keratinocytes that probably explains the more homogeneous morphogenesis of the tissue with SASS-3 or SASS-4 methods.

Another explanation of this delayed formation of the basement membrane is based on the mechanosensitivity of human keratinocytes. Previous researchers have reported relations between substrate stiffness and keratinocyte behavior.^22–24^ At the time of keratinocyte adhesion, and linked to the production method, mechanical properties of the interface between cells and the underlying extracellular matrix could be different in SASS-3 and SASS-4 when compared with SASS-2, and thus, differentially stimulate keratinocytes.

## Conclusions

Our new production methods for SASS allow for the fabrication of high-quality skin substitutes. They are designed for the permanent coverage of full-thickness wounds to replace both the dermis and the epidermis in a single-step grafting procedure. SASS-2, SASS-3, or SASS-4 possesses many desired features such as an autologous epithelium, a *stratum corneum*, a basement membrane at the dermoepidermal junction, a collagen-rich dermal component, mechanical stability, and permanent epithelialization after grafting. A clinical trial assessing the efficacy of SASS for the permanent coverage of full-thickness wounds is currently ongoing in Canada (ClinicalTrials.gov NCTNCT02350205).

## Abbreviation Used

SASS: Self-Assembled Skin Substitute
